# Dipeptidyl Peptidase-4 Inhibitors: A Systematic Review of Structure-Activity Relationship Studies

**DOI:** 10.5812/ijpr-151581

**Published:** 2024-10-29

**Authors:** Maryam Bayanati, Mohammad Ismail Mahboubi Rabbani, Shirin Sirous Kabiri, Bahareh Mir, Elham Rezaee, Sayyed Abbas Tabatabai

**Affiliations:** 1 Department of Food Technology Research, National Nutrition and Food Technology Research Institute, Faculty of Nutrition Sciences and Food Technology, Shahid Beheshti University of Medical Sciences, Tehran, Iran; 2 Department of Medicinal Chemistry, Faculty of Pharmacy, Tehran Medical Sciences, Islamic Azad University, Tehran, Iran; 3 Department of Pharmaceutical Chemistry, School of Pharmacy, Shahid Beheshti University of Medical Sciences, Tehran, Iran

**Keywords:** DPP-4 Inhibitors, Dipeptidyl Peptidase-4, Docking

## Abstract

**Context:**

Dipeptidyl peptidase 4 (DPP-4) is a serine exopeptidase enzyme that hydrolyzes the amide bond at the N-terminal of peptides. This enzyme converts incretins, such as glucagon-like peptide I and glucose-dependent insulinotropic peptide, into their inactive forms, thereby preventing them from stimulating insulin secretion. Numerous studies have confirmed the role of DPP-4 in the pathophysiology of type 2 diabetes, leading to the development of various DPP-4 inhibitors. In recent years, research on DPP-4 inhibitors has expanded significantly, resulting in the creation of both non-peptidomimetic heterocyclic compounds and peptidomimetic scaffolds.

**Evidence Acquisition:**

This systematic review summarizes all recent advances related to DPP-4 inhibitors up to 2024. It begins by outlining the biochemical characteristics of DPP-4 and general pharmacological principles of DPP-4 inhibition, followed by an overview of the latest developments from recent publications. The review provides valuable insights into the pharmacophores necessary for ligand-protein interactions, aimed at understanding the structure-activity relationship of novel DPP-4 inhibitors. Data for this review was collected from sources including ScienceDirect, PubMed, and Scopus.

**Results:**

This review highlights various chemical scaffolds that have been explored in the development of novel DPP-4 inhibitors. It emphasizes scaffolds with significant DPP-4 inhibitory activity, including azoles, azines, sulfonamides, and quinolone motifs. The article also details the structure-activity relationships of newly developed analogs, providing a comprehensive overview of recent advancements in this area.

**Conclusions:**

Despite moderate progress in the development of novel DPP-4 inhibitors, emerging molecular aspects of DPP-4 intervention show great promise for future therapeutic developments.

## 1. Context

Diabetes mellitus (DM) is a rapidly increasing chronic metabolic disorder that poses a significant threat to global health. By 2030, it is projected that diabetes will affect approximately 439 million people worldwide, with developing countries experiencing the highest rates of increase (In 2010, developing countries accounted for 69% of the total cases, while developed nations accounted for 20%) (1). The two primary types of diabetes are type 1 diabetes mellitus (T_1_DM) and type 2 diabetes mellitus (T_2_DM), also known as non-insulin-dependent diabetes mellitus (NIDDM) (2). In T_1_DM, an autoimmune mechanism impairs β-cells, leading to insulin deficiency, whereas T_2_DM is characterized by insulin resistance and impaired glucose regulation, where the peripheral action of insulin is compromised (3). Chronic untreated T_2_DM often leads to complications, such as cardiovascular disease (4), retinopathy (5), obesity (6), nephropathy (7), neuropathy (8), and neurodegenerative disorders (8).

Currently, various hypoglycemic drugs are used to manage T_2_DM, including meglitinides, thiazolidinediones, sulfonylureas, glucosidase inhibitors, and biguanides. These medications reduce hepatic glucose production, enhance insulin secretion, limit glucose absorption, and improve peripheral glucose utilization (9). However, these treatments often come with side effects such as weight gain, digestive issues, and hypoglycemia, making long-term glycemic control challenging (10, 11). As a result, researchers have sought new therapeutic strategies with different mechanisms of action to enhance T_2_DM management (12, 13). 

Twenty-five years ago, dipeptidyl peptidase-4 (DPP-4) inhibitors, commonly known as "gliptins," were introduced through virtual screening (VS) and high-throughput screening (HTS) methods (14). Since their introduction, DPP-4 inhibitors have gained significant attention for more effective hyperglycemia control (15, 16). The first clinical trial on DPP-4 inhibitors was published in 2002, and subsequent randomized controlled trials (RCTs) demonstrated their efficacy, safety, and tolerability (15, 17). Dipeptidyl peptidase-4 inhibitors are orally active and have a long half-life, enabling once-daily dosing. Clinical trials lasting up to 52 weeks have shown a reduction in HbA1c levels of approximately 1% with DPP-4 inhibition (18). Preclinical studies also suggest that DPP-4 inhibitors help preserve β-cell function, potentially slowing the progression of T_2_DM.

Furthermore, DPP-4 inhibitors play a significant role in the regeneration and differentiation of pancreatic cells and are well-tolerated, reducing the risk of hypoglycemia and cardiovascular side effects (15, 19, 20). Clinical placebo-controlled studies and head-to-head comparisons with other glucose-lowering medications have confirmed the efficacy and tolerability of DPP-4 inhibitors (21, 22). The combination of a DPP-4 inhibitor with insulin is particularly attractive because it does not significantly increase the risk of hypoglycemia, as the insulin secretagogue effect is glucose-dependent. Additionally, DPP-4 inhibitors may help protect against hypoglycemia by influencing pancreatic cells (19, 23). 

Dipeptidyl peptidase-4 is widely expressed across various organs and bodily fluids, including the biliary tract (24), kidneys (25), gastrointestinal tract (26), lungs (27), uterus (28), liver (29-32), and immune cells such as T cells, activated natural killer (NK) cells, and activated β cells (33-36). It is a key regulatory enzyme and signaling factor for two major incretin hormones: Glucagon-like peptide-1/2 (GLP-1/2) and glucose-dependent insulinotropic polypeptide (GIP) (37, 38). These hormones contribute to a stronger insulin response to enteral glucose intake compared to intravenous administration (39). Released in response to food intake, these hormones enhance glucose-dependent insulin secretion, playing an essential role in maintaining normal glucose homeostasis (40). 

In addition, these hormones promote β-cell proliferation, boosting their development and differentiation, as well as the transcription and translation of the pro-insulin gene. However, due to rapid degradation by DPP-4, GLP-1/2 and GIP have short half-lives, with 1-2 minutes for GLP-1/2 and 7 minutes for GIP (18, 41). Beyond diabetes, DPP-4 has also been identified as a moonlighting protein involved in the early stages of cancer development (42). Several in vitro and in vivo studies have demonstrated that DPP-4 inhibitors possess antioxidant, anticancer, and proapoptotic properties, particularly against pancreatic and colon cancer cell lines (HT-29). Their notable anticancer effects suggest potential applications as cytotoxic agents in various tumor scenarios.

Several DPP-4 inhibitors with distinct structures have been approved for clinical use, including sitagliptin, alogliptin, linagliptin, gemigliptin, anagliptin, and teneligliptin (22) (Appendix 1 in Supplementary File). This systematic review presents the most recent and critical DPP-4 inhibitor scaffolds, exploring their structure-activity relationship (SAR).

### 1.1. DPP-4 Inhibitors–Mechanism of Action

Glucose-dependent insulinotropic peptide is a 42-amino acid gastrointestinal regulatory peptide primarily produced by Kappa (κ) cells located in the duodenum (43, 44). Glucose-dependent insulinotropic polypeptide is released into the bloodstream after a meal, while GLP-1 is generated by L cells, which are primarily found in the lower section of the small intestine (18, 44). Dipeptidyl peptidase 4 inhibitors prevent the inactivation of GLP-1 and GLP-2, leading to elevated levels of GLP-1 and GLP-2 both after meals and during fasting (45). Animal studies show that DPP-4 inhibition does not improve glucose homeostasis in mice with genetic deletion of GLP-1 and GIP receptors, highlighting the critical role of GLP-1 in the mechanism of action of DPP-4 inhibitors (46). Glucagon-like peptide-1/2, in turn, promotes insulin production, and DPP-4 inhibitors have been shown to enhance acute β-cell function (45, 47, 48).

Vildagliptin has demonstrated an improved insulin response proportional to the rise in glucose levels after meals and an enhanced predicted insulin secretion rate, based on insulin and C-peptide data. Additionally, sitagliptin has been shown to lower the proinsulin-to-insulin ratio (an indicator of β-cell function) and increase the homeostasis model assessment-B index, a measure of insulin production (49, 50). 

Animal studies have also shown that DPP-4 inhibitors improve chronic β-cell function and increase β-cell mass (51-53), although such evidence has not been observed in humans. Another key mechanism by which DPP-4 inhibitors improve glycemic control is through the inhibition of glucagon production, driven by the GLP-1 effect (22). Vildagliptin has been shown to lower the 24-hour glucagon profile when taken for four weeks at 100 mg once or twice daily (47, 54). A recent study further demonstrated that the suppression of hepatic glucose production, measured via the clamp technique using tracer glucose, was accompanied by a reduction in glucagon levels and increased insulin secretion in subjects with type 2 diabetes administered 100 mg of vildagliptin (55). These findings are especially relevant for type 2 diabetes patients, who typically exhibit abnormally high glucagon secretion.

Moreover, DPP-4 inhibitors may improve insulin sensitivity and islet function (56). This has been observed following vildagliptin treatment using both the hyperinsulinemic-euglycemic clamp test and indirect measures of insulin sensitivity (50, 57). This may result from improved metabolic regulation due to DPP-4 inhibition, reduced glucagon levels, and enhanced insulin activity. Unlike GLP-1, DPP-4 inhibitors do not appear to influence gastric emptying, as evidenced by the lack of impact on the rate of glucose absorption following a meal (21). Additionally, vildagliptin did not affect the digestion rate of a tracer-enriched meal (21).

### 1.2. Dipeptidyl peptidase 4 Structure 

**Figure 1.**
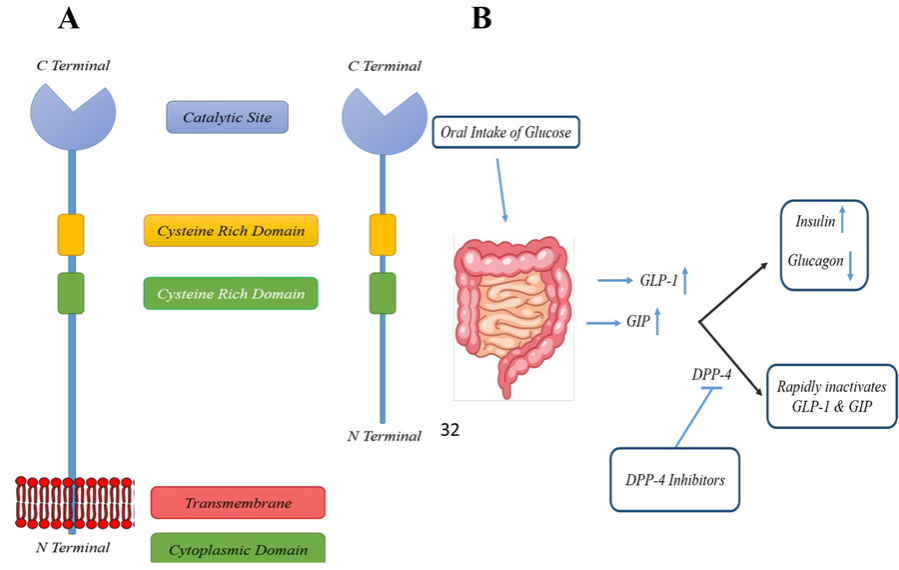
A, the soluble and membrane-bound dipeptidyl peptidase 4 (DPP-4); B, oral glucose intake stimulates the release of glucagon-like peptide-1/2 (GLP-1/2) and GIP, which are rapidly deactivated within 90 - 120 seconds by DPP-4 enzymatic activity.

The DPP family is classified into several enzyme subfamilies, including DPP-4, DPP-7 (proline dipeptidase), DPP-8, DPP-9, fibroblast activation protein α (FAP-α), and prolyl carboxypeptidase (PCP) (25, 58, 59). Dipeptidyl peptidase 4, also known as CD26, is a transmembrane serine protease first discovered by Hopsu-Havu and Glenner in 1966 (60) (Figure 1A). It is widely distributed throughout the body, being expressed on vascular endothelial cells, T lymphocytes, the gastrointestinal tract, hepatobiliary system, kidneys, and pulmonary tract (61, 62).

Human DPP-4 is a 766-amino acid (aa) glycoprotein, consisting of several distinct regions: (1) A cytoplasmic domain (1 - 6 aa), (2) a transmembrane domain (7 - 28 aa), (3) a flexible stalk (29 - 39 aa), (4) a glycosylated region (101 - 350 aa), (5) cysteine-rich regions (55 - 100 aa and 351 - 497 aa), and (6) a catalytic region (506 - 766 aa) (63). Dipeptidyl peptidase 4 exhibits high selectivity for peptides with proline or alanine at the second NH2-terminal position (64). Its extracellular catalytic domain contains a Ser630, Asp708, and His740 triad, which is located at the C-terminal. The extracellular domain also has binding sites for extracellular matrix elements, including fibronectin and adenosine deaminase. Dipeptidyl peptidase 4 cleaves the penultimate prolines or alanines of peptides' N-terminal regions, such as GIP and GLP, effectively inactivating these peptides.

In short, DPP-4 removes two N-terminal amino acids from natural peptides positioned in the penultimate (S_1_) site, such as proline or alanine, thus inactivating GLP-1 and GIP. Both GLP-1 and GIP act on pancreatic β-cells, significantly increasing insulin release. They also suppress glucagon release from α-cells, reducing hepatic glucose production (Figure 1B). As a result, GLP-1 and GIP enhance cellular glucose uptake and contribute to the physiological control of glucose by suppressing hepatic glucose output. Appendix 2 in Supplementary File summarizes some of the most well-known natural substrates of DPP-4 and their corresponding biological functions.

Dipeptidyl peptidase 4 also exists in a soluble form (sDPP-4), which is released from the plasma membrane (65). The soluble form, consisting of 727 aa, lacks the transmembrane and intracellular domains and is found in serum, bile, saliva, and other bodily fluids (66). Importantly, sDPP-4 levels do not correlate with fasting blood glucose levels (67, 68).

### 1.3. Deactivation Mechanism of Glucagon-like Peptide-1/2 by Dipeptidyl Peptidase 4

Intestinal L-cells and K-cells release the physiologically active forms of GLP-1 and GIP, known as active GLP-1 amide and GIP, in response to oral glucose via increased cAMP (69). In addition to stimulating insulin secretion, GLP-1 has been shown to promote pancreatic islet neogenesis, differentiation, and suppression of β-cell apoptosis in rodent models (70). These effects are linked to an increase in β-cell mass. Furthermore, GLP-1 enhances myocardial and endothelial function, offering protective effects against cardiovascular complications associated with hyperglycemia in diabetic patients (71, 72). Numerous studies have demonstrated that intravenously administered GLP-1 reduces the insulin requirement for meal intake in both types of diabetes mellitus (73-77). However, a major challenge in introducing GLP-1 as a novel therapy for type 2 diabetes is its rapid inactivation by DPP-4 (75).

Dipeptidyl peptidase 4 swiftly cleaves the bond between the second and third N-terminal amino acid residues in the active forms of GLP-1 and GIP within 90 - 120 seconds (78), generating biologically inactive forms (79, 80). One strategy to address this limitation is to inhibit DPP-4 activity, thereby preventing the inactivation of GLP-1 (Figure 1B). This is supported by findings that DPP-4 knockout mice exhibit increased glucose tolerance following oral glucose administration. Other bioactive peptides, such as insulin-like growth factor-1, are also potential substrates for DPP-4 (32).

### 1.4. Structure-Activity Relationship (SAR) 

**Figure 2.**
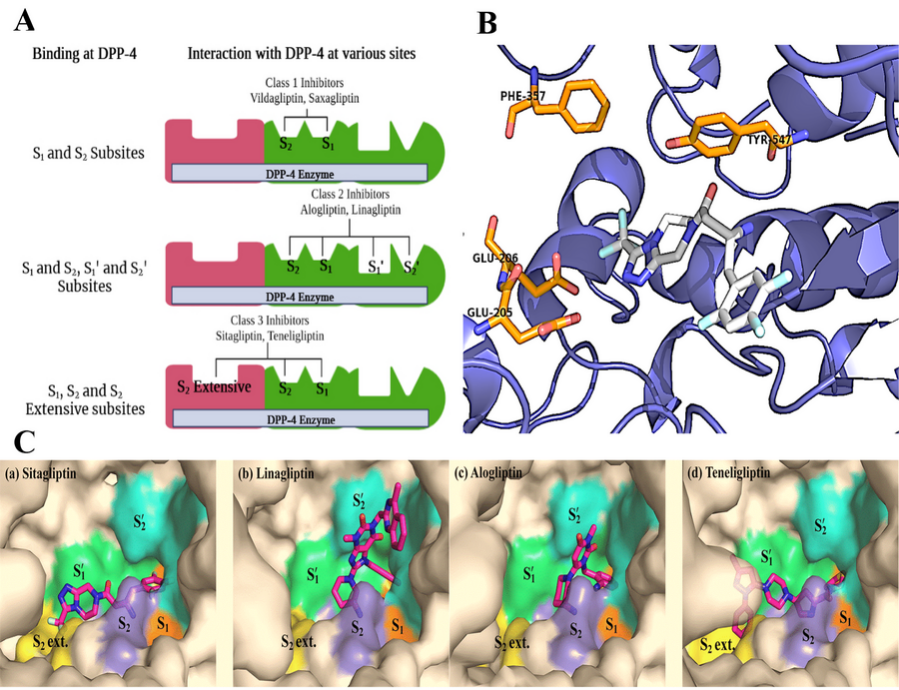
A, graphical representation of the DPP-4 enzyme with binding sites; B and C, DPP-4 inhibitors fitting into the DPP-4 catalytic site, where they interact with critical amino acid residues, including Phe357, Tyr547, Glu205, and Glu206 (87). The figure is partially adapted from "PLOS ONE," Vol. 11, 2016. Copyright (2016, the Protein Society). Reprinted with permission from PLOS, Inc.

Understanding the binding interactions between the DPP-4 enzyme and its inhibitors is key to developing novel DPP-4 inhibitors. Based on the previously elucidated of sitagliptin-derived DPP-4 inhibitors, the DPP-4 binding site consists of two primary pockets, S_1_ and S_2_, and a subpocket known as the S_2_-extensive or S3 pocket (81) (Figure 2A). The S_1_ pocket typically accommodates a substituted aromatic ring or a substituted saturated heterocycle, with the ligand’s main amine forming a hydrogen bond with Glu-205/206 (82). In the S_2_ pocket, an aromatic heterocyclic ring or substituted fused rings are preferred. The hydrophobic S_2_-extensive site should ideally be occupied by an aromatic group, and a spacer linking the S_1_ and S_2_-extensive binding groups is essential (83). By modifying the core structure with the appropriate amine and substituted rings, new DPP-4 inhibitors can be designed (84-86).

It may be hypothesized that there are five distinct binding sites, each associated with a specific class of prominent DPP-4 inhibitors. In the case of class 1 inhibitors, including saxagliptin and vildagliptin, two primary binding sites, S_1_ and S_2_, are coordinated by clusters of amino acid residues. The S_2_ subsite comprises ionized side chains, specifically Arg125, Arg358, Glu205, Glu206, and Arg669, while the S_1_ subsite is composed of polar amino acids such as Ser630, Tyr662, Tyr666, and Asn710. By contrast, linagliptin and alogliptin bind within the enzyme's catalytically active pocket through interactions with both the S_1_/S_2_ and S_1_'/S_2_' binding sites, which are formed by a combination of aromatic amino acids, including Phe357, Tyr547, Tyr631, Tyr666, Trp629, and His740. Finally, class 3 compounds, including sitagliptin and teneligliptin, bind to the enzyme’s active site by anchoring into an accessory binding pocket, referred to as the S_2_ extensive side pocket, which is structured by residues Val207, Ser209, Phe357, and Arg358 (87) (Figure 2C). 

In terms of sitagliptin's binding mode, its trifluorophenyl motif binds with the hydrophobic S_1_ pocket, forming π-bonds with the essential Tyr547 residue (88). The amide group of sitagliptin occupies the S_2_ pocket, creating a salt bridge with Glu205 and Glu206. Additionally, the trifluoromethyl triazole group is positioned within the extensive S_2_ pocket, enabling further bonding interactions (89-92). Dipeptidyl peptidase 4 inhibitors with gliptin-like structures, based on approved and published data, are generally classified into three groups: (a) Compounds with a pyrrolidine as the S_1_-binding segment connected by an aminoacyl spacer, (b) those containing trifluorophenyl derivatives as the S_1_-binding segment with an α-aminobutanoyl spacer, and (c) structures featuring pyrimidine-2,4-dione or its analogs as the S_1_-binding segment. Inhibitors with pyrrolidine/trifluorophenyl moieties as the S1-binding fragment similarly bind to the enzyme’s S_1_, S_2_, and S_2_ extensive domains, whereas the binding mechanism of pyrimidine-2,4-dione analogs differs through additional interactions with the S_1_' and S_2_' pockets (Figure 2A). A summary of findings from previous docking studies of these prominent DPP-4 inhibitors (87) is illustrated in Figure 2B. 

## 2. Evidence Acquisition 

The literature search was conducted using prominent databases, including Google Scholar, Reaxys, SciFinder, PubMed, Scopus, and ScienceDirect. Original research and review articles published within the last ten years were included in the study, while manuscripts without available full text were excluded. The keywords used in the literature search included “design,” “synthesis,” “DPP-4 inhibitors,” “docking,” and “structure-activity relationship.” Initially, 200 papers were evaluated, with duplicates and publications unrelated to the search’s objective discarded. In total, 133 studies were included in the final review.

## 3. Updates on Dipeptidyl Peptidase 4 Inhibitors 

Dipeptidyl peptidase-4 inhibitors are categorized in various ways. Broadly, they can be divided into two general types: Substrate-like inhibitors and non-substrate-like inhibitors. Substrate-like inhibitors often include proline mimetics that occupy the S_1_ site. The S_2_-binding substituent, on the other hand, can occupy the S_2_ site either covalently or noncovalently. Cyanopyrrolidines are one type of inhibitor that docks within the enzyme binding site through two critical interactions: First, a nitrile group forms reversible covalent bonds with the hydroxyl group of Ser630; second, a protonated amino group establishes hydrogen bonds with negatively charged sections of the peptide chain, specifically the Glu205, Glu206, and Tyr662 residues. Non-substrate-like inhibitors, which are non-covalent, contain an aromatic motif that fills the S_1_ pocket and do not mimic the dipeptide nature of DPP-4 substrates. Merck discontinued α-amino acid-containing molecules due to a lack of selectivity and focused instead on selective amino acid piperazine derivatives, which led to the development of sitagliptin, a compound containing piperazine and triazolopiperazine. Additionally, linagliptin, a xanthine-based drug, emerged from another development pipeline and received approval after significant research efforts. Pyrimidinedione derivatives, known for enhanced metabolic stability, led to the potent, selective, and bioavailable DPP-4 inhibitor alogliptin.

Dipeptidyl peptidase inhibitors are also classified according to the subtype of enzyme they are designed to inhibit. Among the various DPP subtypes, DPP-4 is the most clinically significant. Consequently, in the development of clinically relevant DPP inhibitors, DPP-4 is prioritized over other subtypes. In this context, the chemical structures of the most recently developed DPP-4 inhibitors will be reviewed as follows.

### 3.1. Selective Dipeptidyl Peptidase 4 Inhibitors

#### 3.1.1. Triazolotriazine Analogs 

**Figure 3.**
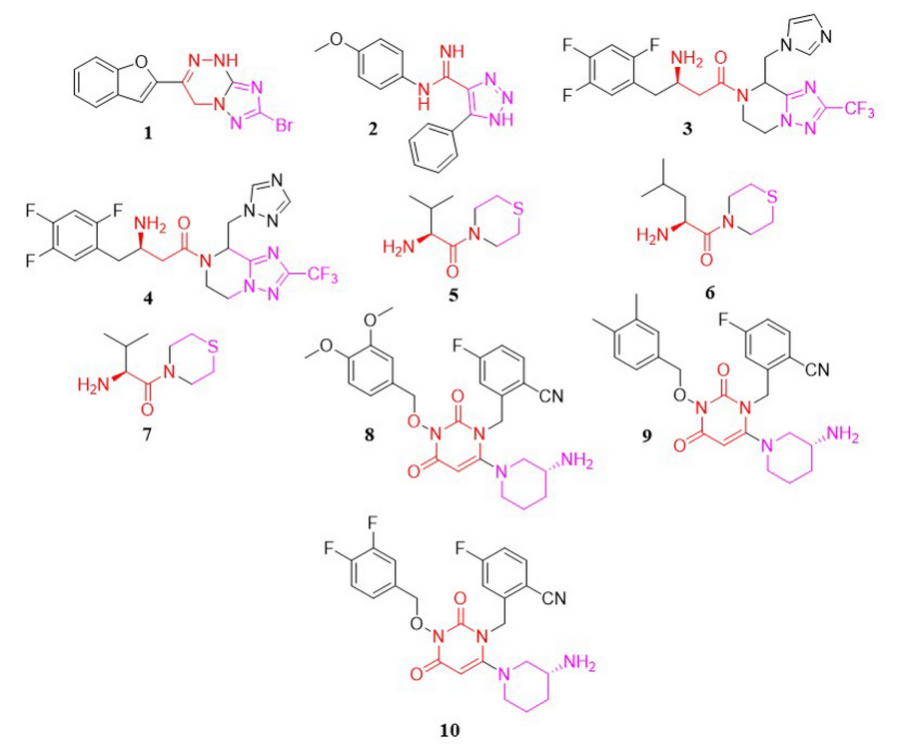
Triazolotriazine, triazole, thiomorpholine, and pyrimidinedione analogs

In a study conducted by Patel et al., a new series of triazolotriazine analogs were developed as DPP-4 inhibitors. The most promising compound was analog 1, with an IC_50_ of 28.05 µM (93) (Figure 3). 

#### 3.1.2. Triazole Analogs 

In another study by Dastjerdi et al., a novel series of 1,2,3-triazole-5-carboximidamide derivatives were synthesized to inhibit DPP-4 catalytic activity. Among these, compound 2 (Figure 3) demonstrated the strongest inhibitory activity, with an IC_50_ value of 6.57 µM (94). 

In 2007, Kowalchick et al. introduced a series of β-amino amides incorporating triazolopiperazine as DPP-4 inhibitors (95). Among the synthesized compounds, molecules 3 and 4 (Figure 3) were identified as the most potent, with IC_50_ values of 2 nM, respectively.

#### 3.1.3. Thiomorpholine Analogs 

Han et al. developed thirteen DPP-4 inhibitor compounds based on the thiomorpholine moiety (96). All of these compounds were evaluated as DPP-4 inhibitors in vitro. The most potent molecules—5, 6, and 7 (Figure 3)—also had their oral antihyperglycemic efficacy assessed. In this compound family, bulkier groups on the carbonyl position enhance inhibitory activity.

#### 3.1.4. Pyrimidinedione Analogs 

In 2018, Li et al. synthesized a novel series of pyrimidinedione derivatives and evaluated their in-vitro DPP-4 inhibitory effects as well as in-vivo antihyperglycemic potential (97). According to the SAR of these compounds, the hydrophobic nature positively influenced DPP-4 inhibitory activity, likely due to their lipid/water partition coefficient. Furthermore, a decreased number of bromine atoms on the phenyl moiety was associated with increased inhibitory activity. Other key factors affecting potency included the alkyl side chain and the presence of the fluorocyanobenzyl group. Among the synthesized and tested compounds, 8 (64.47 nM), 9 (188.7 nM), and 10 (65.36 nM) (Figure 3) showed the highest activity. In vivo antihyperglycemic testing demonstrated that compound 8 effectively reduced blood glucose levels in mice starting from the second week. This compound exhibited potent anti-diabetic properties, significantly lowering blood glucose levels.

#### 3.1.5. Aminoalkyl-Containing Analogs 

**Figure 4.**
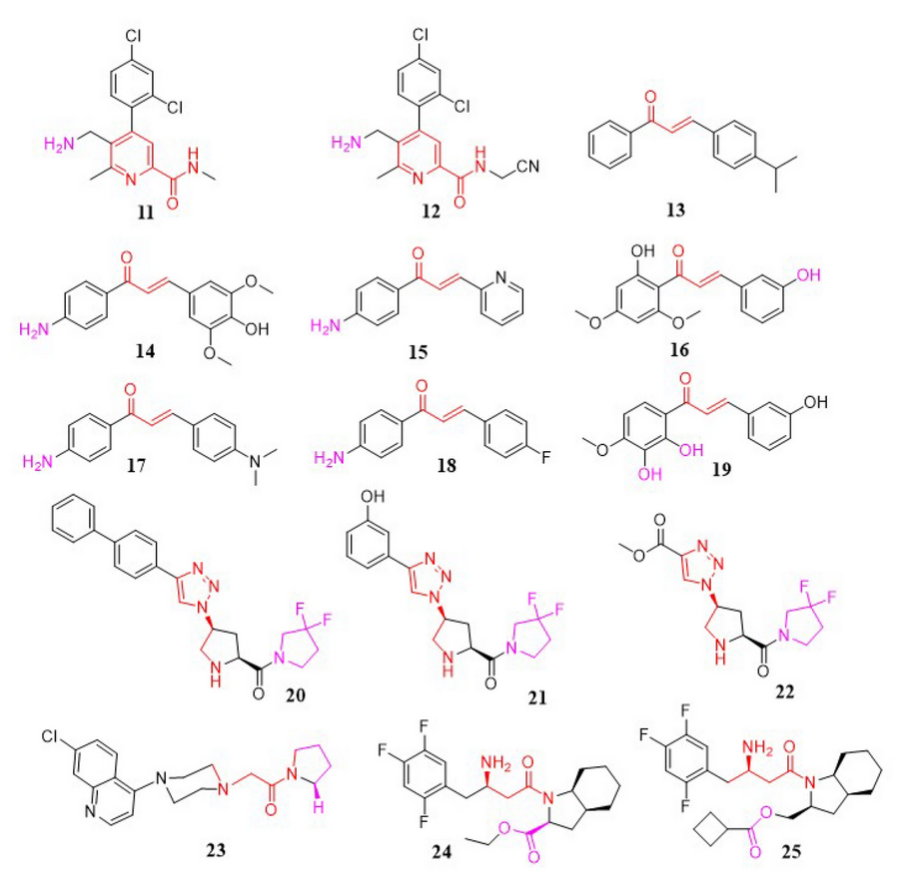
Aminoalkyl-containing, triazolopyrrolidine, piperazinopyrrolidine, and indole aminoethyl carboxamide analogs.

Katarzyna Kaczanowska et al. synthesized a novel collection of aminomethyl-pyridine compounds and evaluated them for DPP-4 inhibitory activity (compounds 11 and 12). The aminomethyl-pyridine moiety demonstrated improved inhibitory effects on DPP-4 in screening results. The significance of the aminomethyl group on the pyridine ring for DPP-4 inhibitors has been highlighted (85); in continuation, this research showed that compounds with aminomethyl in the β-position, rather than the α-position, exhibited lower IC_50_ values and better efficacy. Additionally, the size and position of the amide group in this series were critical; increasing the size of this group and shifting the amide from the α to the β position resulted in a loss of inhibitory activity. The presence of a halogen-substituted phenyl ring was also essential for enhanced potency. Substituting the primary amide with a cyano group and methyl group significantly improved potency. Studies showed that inhibitory activity could increase up to 1000-fold due to interactions between the cyano group and the O-atom of the serine side chain in the catalytic domain of DPP-4 (85) (Figure 4). 

In a study by Rammohan et al., a new series of aminochalcone and hydroxychalcone analogs were developed as DPP-4 inhibitors (98). In-vivo tests using alloxan-induced diabetic rats demonstrated that compounds 13, 14, and 15 (Figure 4) displayed significant anti-diabetic activity, with lower blood glucose levels in diabetic rats compared to control rats. Further docking studies with aldose reductase, DPP-4, PPAR, and glucosidase revealed that chalcones 13, 16, 17, 18, and 19 (Figure 4) inhibited DPP-4 activity more effectively than others. Additionally, these compounds displayed diverse interactions, such as cationic, electrostatic, and hydrophobic connections with critical binding site residues (98).

#### 3.1.6. Triazolopyrrolidine Analogs

Zhang et al. introduced new DPP-4 inhibitors incorporating a triazole moiety. In these compounds, in-vitro results showed that pyridine substitution on the triazole scaffold had a lower inhibitory effect compared to phenyl substitution. In the phenyl-substituted triazole compounds, electron-donating or electron-withdrawing substitutions on the phenyl ring influenced DPP-4 inhibitory activity, with selectivity following the order para > ortho > meta. The 3-hydroxy substitution was the most potent in this group but showed poor selectivity. Additionally, biphenyl and ester substitutions on the triazole ring enhanced effectiveness. In conclusion, compound 20 in the biphenyl-substituted triazole group, as well as compounds 21 and 22 in the acetoxy- and monophenyl-substituted triazole groups, demonstrated the highest potency for inhibiting DPP-4 (99) (Figure 4). 

#### 3.1.7. Piperazinopyrrolidine Analogs 

In a study by Ram Najar Kushwaha et al., a range of compounds was synthesized, among which 1-(2-(4-(7-chloro-4-quinolyl) piperazin-1-yl) acetyl) pyrrolidine (compound 23) was identified as a DPP-4 inhibitor (100). Compound 23 displayed moderate antihyperglycemic activity compared to sitagliptin and showed improvements in insulin resistance reversal and antidyslipidemic properties. As a lead compound, 23 (IC_50_ = 3.73 μM) demonstrates potential for further studies (Figure 4). 

#### 3.1.8. Indole Aminoethyl Carboxamide Analogs

Wang et al. introduced a new series of (R)-3-amino-1-((3aS,7aS)-octahydro-1H-indol-1-yl)-4-(2,4,5-trifluorophenyl) butan-1-one analogs as a novel class of DPP-4 inhibitors. Among these, compounds 24 (IC_50_ = 0.07 µM) and 25 (IC_50_ = 0.07 µM) (Figure 4) showed exceptional inhibitory activity (101). 

#### 3.1.9. Imidazopyridine Analogs 

**Figure 5.**
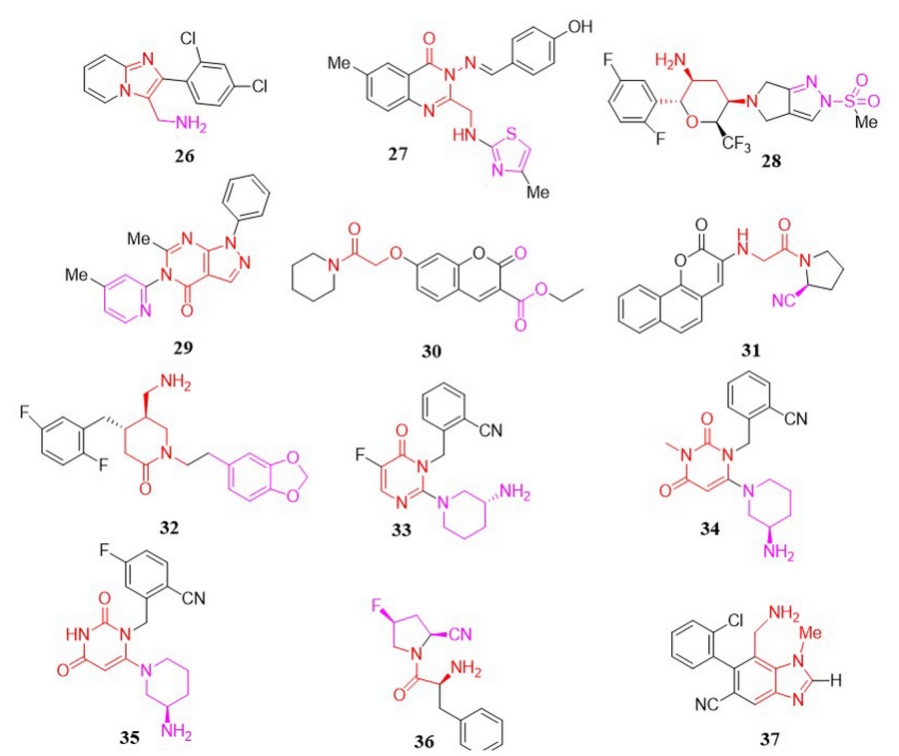
Imidazo [1,2-a] pyridine, thiazoloquinazoline, tetrahydropyran, pyrazolo [3,4-d] pyrimidinone, coumarin, aminomethyl-piperidone, and pyrimidinones/pyrimidinediones, Prolyl-fluoropyrrolidine, and benzimidazole analogs.

Using scaffold-hopping techniques and docking studies, Li et al. designed and synthesized a new series of DPP-4 inhibitors containing an imidazo[1,2-a]pyridine scaffold. Compound 26 emerged as the most potent (92). Based on a receptor-interaction model, structural modifications of the benzene and pyridine rings led to the identification of compound 26 (Figure 5), which includes a 2,4-dichlorophenyl group at two positions, as a potent (IC_50_ = 0.13 µM), selective (DPP-8/DPP-4 = 215, DPP-9/DPP-4 = 192), and in vivo efficacious DPP-4 inhibitor.

#### 3.1.10. Thiazoloquinazoline Analogs 

In 2017, Ali et al. rationally developed a novel series of thiazole-clubbed quinazoline derivatives. These newly synthesized compounds were tested for in-vitro DPP-4 inhibitory activity, with fair to moderate results compared to linagliptin as the reference standard. Compound 27 (IC_50_ = 1.12 nM) was the most promising, exhibiting selectivity for DPP-4 over DPP-8/9. Docking analysis of compound 27 (Figure 5) within the active site of DPP-4 revealed potential binding interactions (102).

#### 3.1.11. Tetrahydropyran Analogs

In research by Zhang et al., a novel series of trifluoromethyl-substituted tetrahydropyran derivatives was designed as potent DPP-4 inhibitors with an extended duration of action. Incorporation of a trifluoromethyl group at the 6-position of the tetrahydropyran ring in omarigliptin (with a (2R, 3S, 5R, 6S) configuration) was found not only to enhance the pharmacokinetic profile in mice but also to maintain robust DPP-4 inhibitory activity. Compound 28 demonstrated exceptional in vivo potency, with an IC_50_ of 4.2 nM, alongside a favorable safety profile, making it a promising candidate for further preclinical research. Clinical trials of compound 28 (Figure 5) suggested its potential as an effective option for biweekly therapy (103).

#### 3.1.12. Pyrazolopyrimidinone Analogs 

A novel series of pyrazolo[3,4-d]pyrimidinones was developed as DPP-4 inhibitors. These molecules were tested for DPP-4 inhibitory activity, with 6-methyl-5-(4-methylpyridin-2-yl)-1-phenyl-1H-pyrazolo[3,4-d] pyrimidin-4(5H)-one (compound 29) (Figure 5) showing the highest activity (IC_50_ = 1.06 µM). Compound 29 also demonstrated potential in vivo blood glucose-lowering efficacy in male Wistar rats (104). 

#### 3.1.13. Coumarin Analogs

Previous literature on coumarin family members indicated that some of these compounds could suppress DPP-4, especially when linked to peptides. Based on this, Soni et al. introduced a new series of small peptide-linked coumarins as DPP-4 inhibitors (105). Docking studies revealed that compounds with aromatic amine residues interact with the large pocket of DPP-4, while compounds with cyclic secondary amine residues bind in the small pocket, similar to Vildagliptin. Although these compounds displayed lower activity than conventional drugs like Vildagliptin and Sitagliptin, two of the newly synthesized compounds showed potential DPP-4 inhibitory effects at a concentration of 10 µM. Notably, compound 30 (Figure 5) exhibited strong activity, with 56.8% inhibition at 10 µM.

Sharma and Soman developed a novel group of structurally similar 3-aminocoumarin derivatives as DPP-4 inhibitors (106). In vitro experiments found compound 31 (Figure 5) to be the most potent, as predicted by in-silico simulations, with an IC_50_ of 3.16 µM. In further research by Soni and Soman., newly developed aminocoumarins and 7-amino-4-methylcoumarin were screened as anticancer agents against A549 lung cancer and MCF-7 breast cancer cell lines using the MTT assay. Compounds containing proline demonstrated notable anticancer activity, with an IC_50_ of 24 nM (Figure 5) against the A549 cell line, compared to the standard drug fluorouracil (IC_50_ = 11.13 μM) (107).

#### 3.1.14. Aminomethyl-Piperidone Analogs 

Jadav et al. developed and tested a variety of new aminomethyl-piperidones as potential DPP-4 inhibitors (108). The optimized analog, compound 32 ((4S,5S)-5-(aminomethyl)-1-(2-(benzo[d] [1,3] dioxol-5-yl) ethyl)-4-(2,5-difluorophenyl) piperidin-2-one), was found to exhibit high levels of DPP-4 selectivity and potency in vitro. Lead compound 32 (Figure 5), with an IC_50_ of 8.5 nM, demonstrated significant and prolonged antihyperglycemic effects as well as an enhanced pharmacokinetic profile.

#### 3.1.15. Pyrimidinone/Pyrimidinedione Analogs 

In 2011, Zhang et al. reported two novel groups of heterocyclic DPP-4 inhibitors (Figure 5), including pyrimidinones (compounds 33, 34) and pyrimidinediones (compound 35), which were identified as active DPP-4 inhibitors (89). In animal models of diabetes, these powerful, selective, non-covalent inhibitors induced lasting reductions in plasma DPP-4 activity and blood glucose levels after a single oral dosage.

#### 3.1.16. Prolyl-Fluoropyrrolidine 

In a study by Wang et al., a new range of 4-fluoropyrrolidine-2-carbonitrile and pyrrolidine-2-carbonitrile derivatives was designed, with compound 36 identified as the most effective and selective DPP-4 inhibitor (109). After an oral glucose challenge, this compound reduced blood glucose levels in ICR and KKAy mice. Compound 36 also exhibited good efficacy in oral glucose tolerance tests, demonstrating great DPP-4 inhibitory activity (IC_50_ = 0.017 μM) and moderate selectivity against DPP-4 in both ICR and KKAy mice. Therefore, compound 36 (Figure 5) was determined to be a reliable lead for designing new compounds with anti-diabetic activities.

#### 3.1.17. Benzimidazole Analogs

Using structure-based drug design, Wallace et al. generated a novel family of non-covalent, benzimidazole-based DPP-4 inhibitors from a small fragment hit (110). Structure-activity relationship studies resulted in the identification of several compounds that are both potent and selective while retaining excellent physical properties and drug-like characteristics. Biological evaluation demonstrated that compound 37 possesses the most potent inhibitory activity, with an IC_50_ of 8 nM (Figure 5). 

#### 3.1.18. Sulfonamide Analogs 

**Figure 6.**
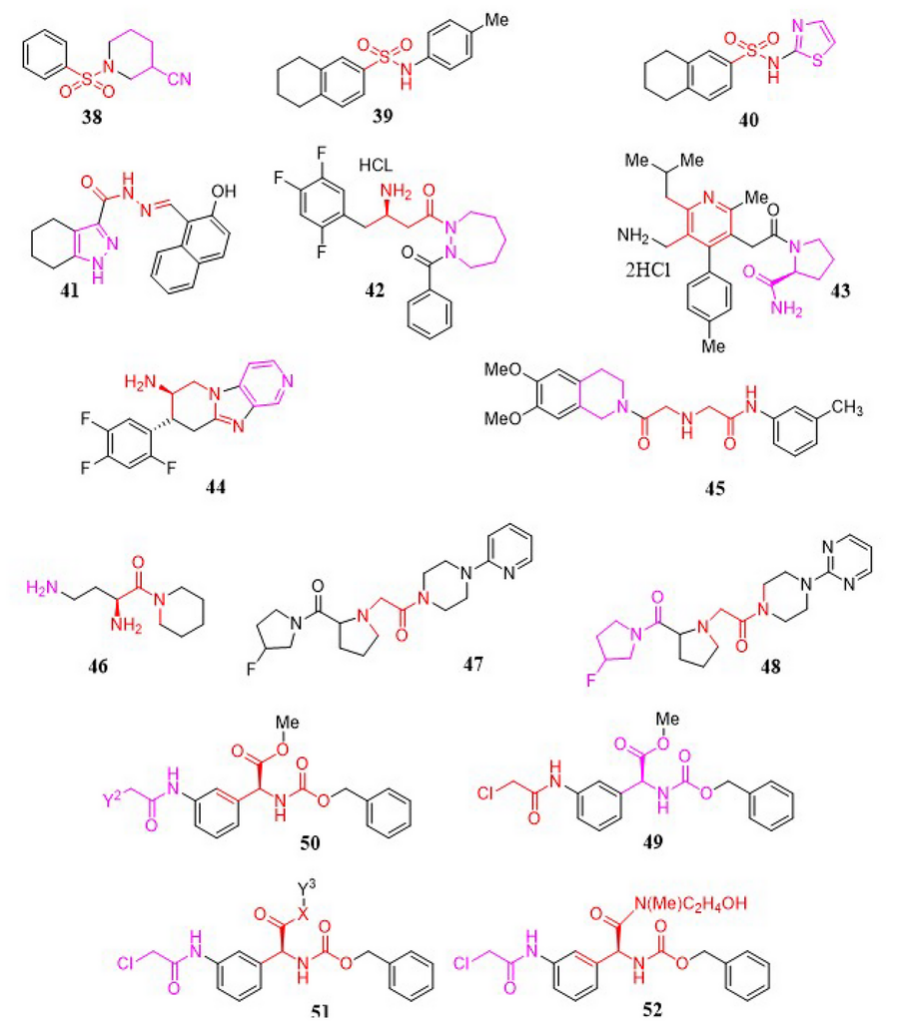
Tetralin-sulfonamide, pyrazole-3-carbohydrazone, β-aminoacyl-containing cyclic hydrazine, nicotinic acid, aminopiperidine-fused imidazopyridine, quinazoline, aminoacyl piperidide, prolyl-fluoropyrrolidine, and L-Phenylglycine analogs.

To discover new DPP-4 inhibitors, Sharma and Soman developed a series of DPP-4 inhibitors containing various sulfonamide-pyrrolidine/piperidine scaffolds, assessing their binding affinities for the enzyme in comparison to Vildagliptin using in silico research. In vitro experiments provided a relative understanding of the binding affinities, revealing that compound 38 displayed similar DPP-4 inhibition in the nM range compared to the reference medication, Vildagliptin. Based on the in vitro studies, it can be inferred that the presence of a nitrile group at the S_1_ site is necessary for DPP-4 inhibition. Ultimately, molecule 38 (Figure 6) was determined to be the most potent agent, with an IC_50_ of 41.17 nM (111). While proline mimetics have been shown to inhibit DPP-4, an unexpected finding in this study was that piperidine-3-carboxylic acid exhibited five-fold greater efficacy than the compound derived from L-proline. Furthermore, the presence of amide functionality at the S_1_ site was found to be undesirable, as it resulted in very poor inhibition of the enzyme. According to the results of this investigation, N-substituted glycine with 2-cyanopyrrolidine at the S_1_ site and sulfonamide derivatives at the S_2_ site effectively inhibited DPP-4.

Abd El-Karim et al. provided a novel series of tetralin-sulfonamide derivatives as DPP-4 inhibitors and anti-diabetic agents (112). A considerable hypoglycemic effect, along with DPP-4 suppression ability in reference to sitagliptin, was exhibited by the majority of the compounds. The most promising compounds, 39 and 40 (Figure 6), showed an IC_50_ of 2.80 nM against DPP-4 catalytic activity, with 20 - 40-fold selectivity over the DPP-8 and DPP-9 isozymes.

#### 3.1.19. Pyrazole-3-Carbohydrazone Analogs 

A series of new DPP-4 inhibitors featuring the pyrazole-3-carbohydrazone structure (39) were identified in work conducted by Deyan Wu et al. using an integrated strategy that combined structure-based virtual screening, chemical synthesis, and bioassay (Figure 6) (113). Following a virtual screening of the SPECS database, along with enzymatic activity experiments, 17 novel compounds were created. DPP-4 inhibitory properties were discovered in nine of these compounds. Ligand binding models provided logical explanations for SAR correlations. The best pharmacophore model was developed with eight DPP-4 inhibitors, featuring one hydrogen bond donor (HBD), one hydrogen bond acceptor, and two hydrophobic characteristics. The docking models and pharmacophore mapping results were consistent with the pharmacological findings.

#### 3.1.20. Cyclic Hydrazine Analogs 

In a study performed by Ahn et al., a collection of β-aminoacyl-containing cyclic hydrazine derivatives was introduced as novel DPP-4 inhibitors (114). Subsequent assay studies indicated that (R)-3-amino-1-(2-benzoyl-1,2-diazepan-1-yl)-4-(2,4,5-trifluorophenyl) butan-1-one (40) possesses strong in vitro activity, favorable selectivity, and in vivo efficacy in mouse models (Figure 6). 

#### 3.1.21. Nicotinic Acid Analogs

In 2011, Miyamoto et al. designed a nicotinic acid derivative as a new DPP-4 inhibitor (115). Focusing on the SAR analysis, Arg125 was selected as a potential amino acid residue for strong inhibitory activation. Following this, a new collection of 3-pyridylacetamide analogs with an additional hydrogen-bond acceptor was designed, which could preferentially engage in bidentate interactions with Arg125. The dihydrochloride of 1-[[5-(aminomethyl)-2-methyl-4-(4-methylphenyl)-6-(2-methylpropyl) pyridin-3-yl]acetyl]-L-prolinamide (41) was identified as showing selective inhibitory activity against DPP-4, capable of interacting with the guanidino group of Arg125 in a bidentate manner (Figure 6). 

#### 3.1.22. Tricyclic Imidazopyridine Analogs 

A viable asymmetric synthesis of a new aminopiperidine-fused imidazopyridine DPP-4 inhibitor has been established (116). The construction of the functionalized piperidinone scaffold in a one-pot manner was facilitated by a novel three-component cascade coupling with a chiral nitro diet, which was easily obtained through a substantially enantioselective Michael addition of dimethyl malonate to nitrostyrene. The cis-piperidinone was epimerized to the required trans isomer using a base-catalyzed, dynamic crystallization-driven procedure, which was subsequently crystallized from the raw reaction mixture with high yield and purity. The allylamide intermediate was isomerized in the presence of RhCl_3_ without any epimerization of the acid/base-labile stereogenic center adjacent to the nitro group on the piperidinone ring. The undesirable enamine intermediate was reduced to 0.5% by using a minimal quantity of HCl generated from RhCl_3_. Ultimately, a Cu(I)-catalyzed coupling-cyclization enabled the formation of the tricyclic structure of the potent DPP-4 inhibitor 44 (Figure 6) (116).

#### 3.1.23. Isoquinoline Analogs

In a study conducted by Xing et al., various factors were integrated to discover a novel group of DPP-4 inhibitors (117). HWL-405 and HWL-892 were previously identified as two notable compounds that exhibited stable and elevated efficacy at all levels of virtual screening. The target derivatives were devised and synthesized accordingly. Based on empirical findings, compound 45 showed the best inhibitory activity against DPP-4 in vitro, with an IC_50_ value of 78 nM. This compound reduced blood glucose levels in a dose-dependent manner in normal male Kunming mice (Figure 6). 

#### 3.1.24. Aminoacyl Piperidides 

Various new organic peptidomimetic scaffolds were primarily explored as novel dipeptidylpeptidase inhibitors by Senten and coworkers (44, 118). This study began with a SAR analysis on aminoacyl pyrrolidides. The logical identification of S_1_ and S_2_ building blocks led to the discovery of effective DPP-2 inhibitors, which were characterized by their high selectivity for DPP-2 over DPP-4. The most promising selected inhibitor was Dab-Pip (Figure 6). 

#### 3.1.25. Prolyl-Fluoropyrrolidine Analogs 

A range of prolyl-fluoropyrrolidine derivatives was synthesized as DPP-4 inhibitors by Sharma et al. (119). Compounds 47 and 48 (Figure 6), with IC_50_ values of 0.83 and 0.43 μM, respectively, which possess aryl-substituted piperazines with acetamide linkers, showed the best activity as DPP-4 inhibitors. Both compounds exhibited significant blood glucose reduction in streptozotocin-induced diabetic rats at a dose of 10 mg/kg.

#### 3.1.26. L-Phenylglycine Analogs 

Liu et al. reported a group of L-phenylglycine analogs to investigate their DPP-4 inhibitory activities (120). Following biological evaluation, molecule 49 (Figure 6) was selected as the hit compound from which compounds 50 and 51 were designed. Analog 52 was identified as the most active analog based on the PPRE relative activities. 

#### 3.1.27. α-Amino Pyrrole-2-Carbonitrile 

**Figure 7.**
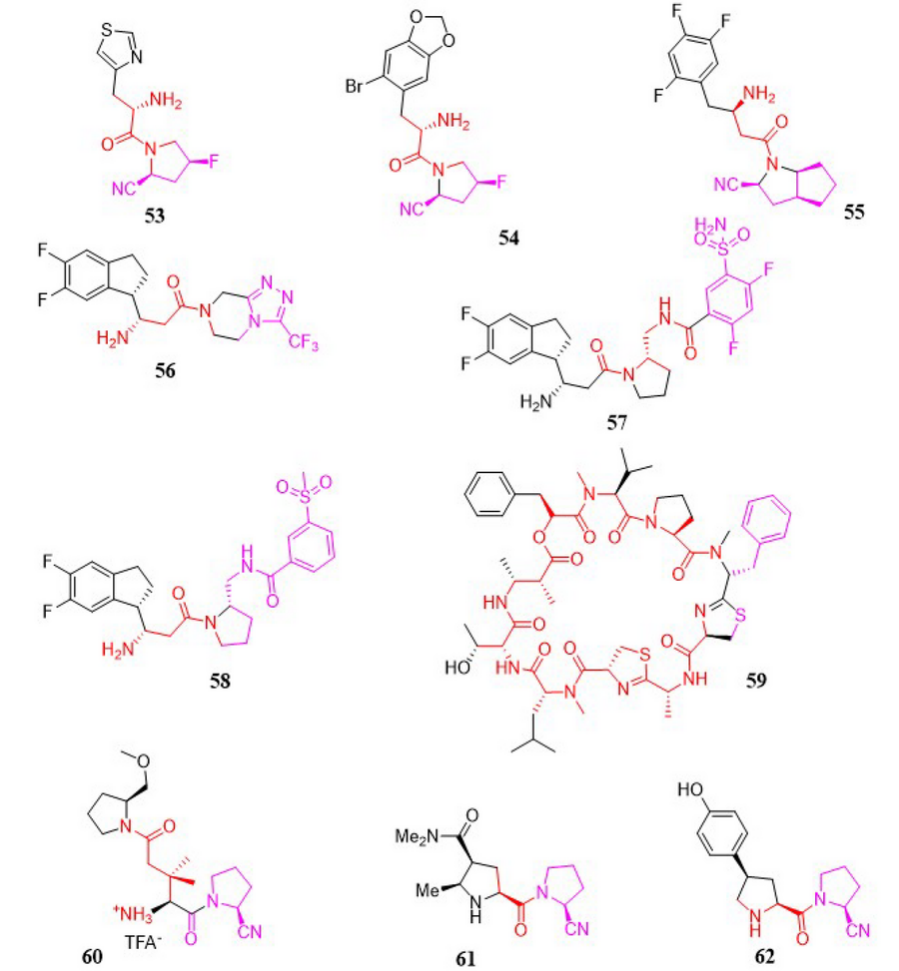
α-amino pyrrole-2-carbonitrile, grassypeptolides, glutamic acid, prolylpyrrolidine analogs, and non-selective dipeptidyl peptidase inhibitors.

A novel group of heteroaromatic moieties substituted with α-amino pyrrole-2-carbonitrile analogs was developed as DPP-4 inhibitors. All newly synthesized analogs demonstrated good inhibitory activities in the range of 0.004 to 113.6 µM. Among these, compounds 53 (IC_50_ = 4 nM) and 54 (IC_50_ = 10 nM) exhibited high DPP-4 inhibitory activities, good efficacy, and selectivity in an oral glucose tolerance test in ICR mice (Figure 7). Moreover, compounds 55 and 56 demonstrated moderate pharmacokinetic properties (121). 

In a study by Ji et al., a novel group of β-amino pyrrole-2-carbonitrile analogs was developed as selective DPP-4 inhibitors, with compound 55 (Figure 7) identified as the most potent, exhibiting an IC_50_ value of 0.01 µM. All newly synthesized analogs demonstrated good inhibitory activities within the range of 0.004 to 113.6 µM (122). 

Optimization of a novel series of fused β-homophenylalanine DPP-4 inhibitors was reported in detail by Jiang et al., leading to the development of β-homophenylalanine analogs containing pyrrolidin-2-yl methyl amides (123). It was found that meta-substitution of the phenyl ring with a sulfamoyl group enhanced the DPP-4 inhibitory activity of the lead compound 56 (Figure 7). Following in vitro tests, compound 57 (Figure 7) was shown to be the most potent DPP-4 inhibitor, with an IC_50_ of 0.87 nM. Meanwhile, in vivo studies revealed that compound 58 (Figure 7) exhibited efficacy comparable to sitagliptin at a dosage of 10 mg/kg.

#### 3.1.28. Grassypeptolides

Natural products produced by marine cyanobacteria are frequently significantly altered peptides and depsipeptides with the ability to function as protease inhibitors. To develop novel protease inhibitors with high activity and selectivity, grassypeptolide 59 was synthesized by Jason C. Kwan and shown to inhibit DPP-8 preferentially over DPP-4 in activated T-cells (124), highlighting DPP-8's potential significance in the immune system. Such properties were also observed in Jurkat cells, where grassypeptolides were found to block DPP activity in the cytoplasm. In silico docking studies indicate two probable binding sites for grassypeptolides: The active site of DPP-8 and one of the entrances to the internal cavity (Figure 7). 

#### 3.1.29. Glutamic Acid Analogs 

In 2009, a research team led by Ting-Yueh Tsai developed a new range of glutamic acid analogs as DPP-4 inhibitors. Through biological assays, compound 60 was noted for possessing 3,3-dimethyl substituents at the β-position of the S_2_ site glutamic acid, with an IC_50_ of 14 nM and an outstanding selectivity profile over DPP-8 (IC_50_ = 14 µM), FAP (IC_50_ > 20 µM), and DPP-2 (IC_50_ > 20 µM) (125). The in vivo actions of compound 60 (Figure 7) were also established, including the reduction of plasma DPP-4 activity and the control of rising blood glucose levels.

#### 3.1.30. Prolylpyrrolidine Analogs 

Takashi Kondo et al. examined the DPP-4 inhibitory activity and the duration of ex vivo activity of a variety of (4β-substituted)-L-prolylpyrrolidine analogs lacking the electrophilic nitrile function. The potency and duration of action of an N-(3-phenyl-1,2,4-thiadiazol-5-yl) piperazine analog, discovered through high-speed analog synthesis, were enhanced via structural optimization. A representative compound 61 (Figure 7) was tested for its impact on plasma glucose levels following the OGTT (126). 

A series of (4-substituted prolyl) prolinenitriles as DPP-4 inhibitors were evaluated in a study by Kondo and coworkers (127). Strong and stable inhibitory activity was observed in 4β-[4-(hydroxyphenyl) prolyl] prolinenitriles, which demonstrated a long duration of action. It was found that the metabolic formation of the corresponding phenol glucuronates contributed to this prolonged action. The final biological evaluation confirmed that compound 62 (Figure 7) possessed the highest enzyme inhibitory activity, with an IC_50_ of 2.5 nM.

### 3.2. Non-Selective Dipeptidyl Peptidase Inhibitors

The existence of a DPP-8 selective inhibitor would be extremely beneficial for untangling the biological activities of DPP-8 and DPP-9, as well as for disambiguating the biological effects of non-selective DPP inhibitors, which have mostly been attributed to the inhibition of DPP-4's action. One of the first dedicated investigations aimed at uncovering modification sites in the topology of a representative DPP-8/9 inhibitor capable of imparting selectivity for DPP-8 over DPP-9 was conducted by Van Goethem et al. in 2011 (128). The cell-permeable DPP-8/9 inhibitor 7 was chosen as a lead compound and deconstructed into different substructures that were modified individually to assess their ability to contribute to selectivity. The results, along with previous reports, substantially narrowed down the most likely sites for DPP-8 selectivity, indicating modification points in DPP-8/9 inhibitors corresponding to topologically comparable areas of space. The challenging nature of the task was considered in light of the strikingly identical amino acid sequences between the active regions of these enzymes. The analogs of 7 created in this manner were tested for DPP-8 and DPP-9 activity, as well as for activity against DPP-4 and DPP-2. All inhibitors included 5-fluoroisoindoline and isoindoline as the optimal S_1_ residues, a trait previously explored in depth by earlier studies. The SAR analysis revealed that an aspartyl residue at the S_2_ position was superior for inhibitory efficacy compared to succinyl and glutamyl moieties. Furthermore, a piperazin-1-yl moiety containing a large acyl or alkyl substituent at the 4-position was identified as the most promising candidate to complement the optimal S_2_ aspartyl residue. Compared to the lead structure 7, nearly all produced compounds were found to be good selective inhibitors for DPP-4. However, compounds in which the S_2_-supplementing piperazine group was either replaced with a 3-aminopyrrolidine counterpart or eliminated regained DPP-2 binding ability. The methyl piperazine analogs of 7 exhibited the highest selectivity for DPP-8 over DPP-9, with nearly an order of magnitude difference. While this level of selectivity for DPP-8 remains low, C-alkylation of the piperazine ring was found to be the only modification method that improved DPP-9 uncoupling relative to the lead compound. Additional structural alterations are currently being researched to develop molecules with the highest affinity and selectivity for DPP-8. By limiting the most probable DPP-8-selectivity imparting modification points in DPP-8/9 inhibitors to areas of space that are topologically similar to the piperazine ring, this strategy may serve as a useful platform for developing future selective DPP-8 inhibitors, including novel chemotypes. The biological evaluation of the newly synthesized chemical agents demonstrated that molecule 63 possesses the most potent enzyme inhibition activity, with an IC_50_ of 17.7 µM (Figure 7). 

## 4. Discussion 

Despite several methods for treating T2DM, finding the optimal therapy for T2DM remains a challenge. As discussed earlier, DPP-4 inhibitors, which work by suppressing incretin function, are promising agents for treating diabetes with fewer adverse effects. In this regard, the substantial volume of data concerning the SAR of DPP-4 inhibitors provides valuable insights into the design of more effective anti-diabetic medications. Recent research on in-silico methodologies, such as virtual screening and homology modeling, could be used to develop innovative DPP-4 inhibitors. If we categorize DPP-4 inhibitors, there are three primary groups to consider. The first group includes ligands that possess a pyrrolidine motif as an S_1_-binding segment with an α-aminoacyl spacer. We will now discuss the SAR of this category of inhibitors in more detail.

The S_1_ domain is believed to be a critical site for DPP-4 inhibitor interaction. Many DPP-4 inhibitors contain five-membered heterocyclic rings as proline mimetics at the S_1_ location. For instance, cyanopyrrolidine and thiazolidine moieties bind to DPP-4's S_1_ pocket, as seen in vildagliptin and saxagliptin. The nitrile forms a covalent imidate with the hydroxyl group of Ser630, which subsequently forms a hydrogen bond with the hydroxyl side group of Tyr547. Additionally, incorporating cyclopropane into cyanopyrrolidine facilitates hydrophobic interactions with Tyr666 in the S_1_ domain, resulting in enhanced inhibitory activity. It was found that when the amide or carboxyl groups are substituted for the cyano group, the inhibitory activity decreases significantly. This decrease may be due to the preferential interactions of pyrrolidine carbonitriles with the critical residues of the S_1_ pocket.

Non-fluorinated pyrrolidine analogs are found to be less potent than their fluorinated counterparts. Within the S_1_ pocket, the difluoropyrrolidide fragment establishes hydrophobic interactions. A hydrogen bond forms between one of the pyrrolidine fluorines and either Ser630 or Tyr631. Clearly, hydrophilic components, such as hydroxypyrrolidine, are not tolerated by the lipophilic S_1_ domain. The carbonyl group of the linker was discovered to form a hydrogen bond with Asn710, whereas the primary or secondary α-amino group forms salt bridges with Glu205 and Glu206. Therefore, the primary or secondary amino group in the linker is essential for inhibitory potential.

Pyrazolopyrimidine, another similar aromatic core structure, forms π-π interactions with the phenyl substituent of Phe357. Due to steric effects or planarity distortion, the 7-monosubstitution and 5- and 7-disubstitution of pyrazolopyrimidine tend to diminish inhibitory activity. In summary, the π-interaction with Phe357 and the negatively charged environment surrounding Arg358 significantly enhance the effectiveness of DPP-4 inhibitors. Indeed, greater occupancy of the hydrophobic cavity between Arg358 and Ser209 potentiates inhibitory activity.

The second group, featuring a trifluorophenyl motif as S_1_, along with a β-aminobutanoyl spacer, can be discussed similarly. Some of the most prominent approved DPP-4 inhibitors containing the trifluorophenyl moiety as the S_1_ component and the β-aminobutanoyl linker include evogliptin, sitagliptin, and gemigliptin. Trifluorophenyl analogs have been found to possess higher potency than difluorophenyl analogs. The piperidinone also engages in the S_1_ pocket, where the fluorine on the upstream side forms a hydrogen bond with Tyr631, while the fluorine on the downstream piperidine makes a hydrophobic contact with Tyr666 and Tyr662. In the S_2_ subsite, the β-amino group of the β-aminobutanoyl linker forms hydrogen bonds with the hydroxyl oxygen of Tyr662 and the carboxyl oxygens of Glu205 and Glu206. The carbonyl group of the aminoacyl moiety establishes a hydrogen bond with Tyr547. In some newly developed DPP-4 inhibitors, the extensive S_2_ subsite is occupied by the triazolopyrazine moiety with a trifluoromethyl substituent. Among β-aminoacyl-containing DPP-4 inhibitors featuring an amino acid moiety, the valine derivative exhibits both in vitro and in vivo potency.

But what about the third group? A versatile collection of pyrimidine-2,4-diones has been reported as DPP-4 inhibitors, with trelagliptin, linagliptin, and alogliptin being well-established members of this group. Pyrimidine-2,4-diones function as the S_1_' segment, and the S_1_ and S_2_ domains interact with the pockets of S_1_' and/or S_2_' of the enzyme as well. This core scaffold and its analogs form π-π interactions with Tyr547, and their conformation is altered in the S_1_' pocket.

DPP-4's S_2_ pocket accommodates a wide range of lipophilic rings, with only a slight decrease in effectiveness for very small three-membered or large twelve-membered rings. Linear chain fragments exhibit lower activity than structures possessing a cyclic moiety at the terminal amine. Furthermore, the hydroxyl group on the adamantyl molecule forms hydrogen bonds with Ser209 and Tyr547. Compared to adamantane, lipophilic substituents on the adamantyl molecule reduce inhibitory efficacy. Substitutions on the adamantyl 3-hydroxyl group, such as carbamate or ester groups, have been shown to lead to diminished inhibitory potential.

It is demonstrated that activity is more dependent on the variation of the substituents compared to the core. In other words, derivatives with different substituents on a single core have a greater impact and display higher variations in potency compared to changes in the core structure. The NH group of Tyr631 forms a hydrogen bond with the carbonyl oxygen of pyrimidine-2,4-diones. The cyanobenzyl group at the N1 position of the pyrimidine-2,4-dione compounds is strategically placed in the S_1_ domain, and the nitrile group forms a hydrogen bond with Arg125. Finally, a salt bridge is created between Glu205/Glu206 and the amino group at the C6 position of the aminopiperidine moiety in the pyrimidine-2,4-dione. In summary, it is noted that the interaction with the Glu205/206 pair and the S_1_ pocket is crucial for inhibitory potency. It is shown that the binding free energy of DPP-4 inhibitors is significantly high when the inhibitors interact with the negatively charged Glu205/206 pair and the S_1_ lipophilic pocket. Newer inhibitors should include hydrogen-bond donor or positively ionizable groups that can interact with the Glu205/206 pair in the N-terminal recognition region, as well as hydrophobic components to engage with the S_1_ pocket.

### 4.1. Conclusions

In summary, this review provides a mixed SAR analysis and experimental data regarding the activity of previous gliptins and gliptin-like DPP-4 inhibitors within the human body. This briefing led to the establishment of three major protein-ligand interaction fingerprints that may serve as a source of inspiration for researchers seeking to enhance evidence-based inhibitors further.

ijpr-151581-Supplementary-file.pdf

## Data Availability

The dataset presented in the study is available on request from the corresponding author during submission or after publication.
