# Supplementary material for: Dipeptidyl Peptidase-4 Inhibitors: A Systematic Review of Structure-Activity Relationship Studies
Source: Iran J Pharm Res. 2024 Oct 29;23(1):e151581. doi: 10.5812/ijpr-151581 (PMC11892788; doi:10.5812/ijpr-151581)
Supplement: ijpr-151581-Supplementary-file.pdf [file ijpr-151581-Supplementary-file.pdf]

AppendiDipeptidyl peptidase 4 inhibitors: a review of structure-activity relationship studies

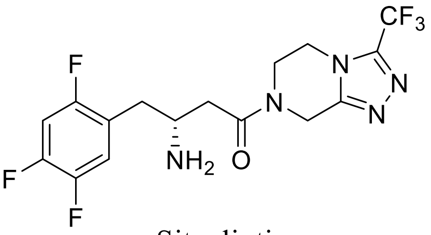

Sitagliptin

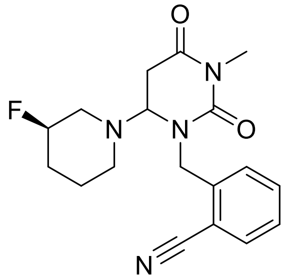

Alogliptin

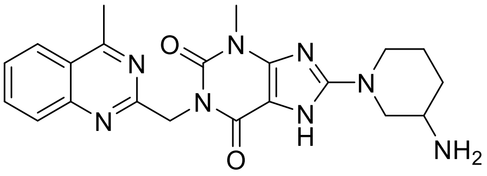

Linagliptin

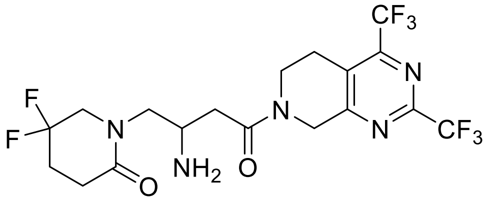

Gemigliptin

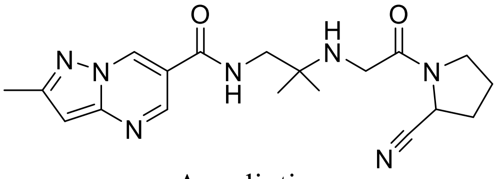

Anagliptin

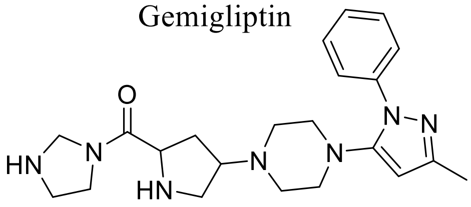

Teneligliptin

.

Appendix 1. The chemical structures of well-known DPP-4 inhibitors

Appendix 2. Prevalent DPP-4 sequences with Xaa-Pro/Xaa-Ala bonds and their relative actions.

**Substrate**	**N-terminal Site**	**Biological Function**
GLP-1	His-Ala-Glu-Gly-Thr-Phe	+ Insulin Release; - Glucagon Output
GIP	Tyr-Ala-Asp- Gly-Ser-Phe	+ Insulin secretion; - Glucagon Output
CXCL-12	Lys-Pro-Val	Chemotaxishttp://en.wikipedia.org/wiki/Chemotaxis of lymphocytes; Angiogenesis
NPY	Tyr-Pro-Ser	Vasoconstriction; Lipogenesis
